# Foot burns and diabetes: a retrospective study

**DOI:** 10.1186/s41038-015-0024-6

**Published:** 2015-12-30

**Authors:** E. Lawrence, F. Li

**Affiliations:** Concord Hospital Burns Unit, Rhodes, Australia

**Keywords:** Diabetes, Foot, Burns, Age, Regrafting, Wound healing

## Abstract

**Background:**

Diabetes in conjunction with a foot burn can compound the challenges in wound healing; however, the impact of diabetes on outcomes of patients with foot burns has not been examined.

**Methods:**

A retrospective notes audit was conducted at the Concord Hospital Burns Unit for patients with foot burns who were admitted from 1^st^ January 2012 to 31^st^ December 2013. Data were collected for 15 subjects with foot burns and diabetes and 18 subjects with foot burns and no diabetes as a control group. Subjects were matched for percentage total body surface area of burns.

**Results:**

The mean inpatient and total lengths of stay for the diabetic group were 21.27 days and 64.80 days, which were significantly longer (*P* = 0.090 and *P* = 0.054) than the 9.61 days and 30.56 days in the control, based on a significance level of 0.10. The diabetic group was significantly older (*P* = 0.001), at 56.60 years versus 39.44 years in the control. Significantly (*P* = 0.033) more patients with diabetes were not working (*n* = 12/15 or 80.00 % versus *n* = 7/18 or 38.89 %) compared to the control. The diabetic group had higher rates of regrafting (*n* = 3/15 or 20.00 % versus *n* = 1/18 or 5.55 %) than the control and significantly (*P* = 0.013) more amputations (*n* = 5/15 or 33.33 % versus *n* = 0 or 0.00 %) compared to the control. Fewer patients with diabetes were prescribed pressure garments (*n* = 2/15 or 13.33 % versus *n* = 9/18 or 50.00 %), which was significant (*P* = 0.034). The increased age of patients in the diabetic group correlates with results from other studies. Healing time may be reflected by total length of stay, which was more than double for patients with diabetes, increasing demand and cost of inpatient and outpatient services.

**Conclusion:**

This study highlights the importance of recognizing the potential for poorer outcomes for patients with diabetes and indicates the need for more burn prevention education and promotion in this ‘at risk’ patient group.

## Background

Diabetes can predispose a patient to foot burns and prolong healing time. Reduced peripheral sensation and circulation can affect recognition of injury and delay presentation to a burn clinic for wound management [1, 2]. It is recommended that patients with foot burns, or burns and a pre-existing medical condition that could adversely affect patient care and outcomes, be referred to a specialized burn unit for appropriate management [3]. The Concord Hospital Burns Unit is part of the New South Wales (NSW) Severe Burn Injury Service and is one of the largest adult burns services in Australia. It has been observed in the clinical setting that burns to the feet can pose a challenge for healing and can cause difficulty walking. Diabetes as a comorbidity is routinely considered by staff when progressing mobility and in planning a patient’s discharge from hospital, especially with burns that involve the feet. Appreciating how the location of burns, the specialized anatomy of skin and the biomechanics of walking can impact the process of healing is important in managing foot burns appropriately. Additionally, understanding the pathophysiology of diabetes and the potential for poorer outcomes can assist health professionals to achieve optimum management of this ‘at risk’ patient group.

Burns to the feet can cause significant morbidity, pain and difficulty in walking due to the location of injury and the specialized anatomy of skin on the sole or dorsum of the foot. Glabrous skin on the sole of the foot is highly specialized and is difficult to reconstruct after a severe burn injury. It has a thicker epidermis, a compact, less elastic dermis and is cushioned with a fat pad. This makes it more durable and able to withstand force, pressure and shearing [4]. The skin on the dorsum of the foot is thin with minimal subcutaneous tissue. It protects and allows for gliding movement of extensor tendons. Severe burns in this area of the foot can be difficult to manage due to risk of tendon and bone exposure, tendon damage and scarring. Preventing complications during healing is important to preserve normal biomechanics and reduce morbidity after severe burns [5].

Foot burns can be managed conservatively with bed rest, foot elevation, regular wound cleansing and dressings, with the aim of reducing edema, avoiding infection, optimizing wound healing and preventing conversion to a deeper burn [2, 6]. Surgical management may be required, including excision of eschar, skin grafting, reconstructive surgery or amputations. Whether managed conservatively or surgically, off-loading pressure on the area affected is important to promote wound healing. Prolonged bed rest, protective footwear or resting splints may be used to protect granulating tissue and assist healing [7].

Diabetes in conjunction with a foot burn can compound the challenges in wound healing. It is well documented that patients with diabetes are more susceptible to wound breakdown, infections and skin graft loss [1]. Diabetes is a significant risk factor for slow wound healing because it is associated with impaired blood flow, peripheral neuropathy and altered function of the immune system [1, 7]. Peripheral blood flow may be impaired due to atherosclerotic changes in distal arteries or increased blood viscosity from hyperglycemia, which can lead to ischemic extremities. Peripheral neuropathy affects motor and sensory nerve function [8]. Vascular insufficiency and peripheral neuropathy can affect the ability to recognize pain or injury to a healing wound and can predispose a patient with diabetes to unnecessary mechanical trauma in weight bearing activities during the healing phase. This can lead to repeated wound breakdown and delayed healing. Autonomic function can also be impaired in patients with diabetes, including function of sweat glands, which can cause dry skin susceptible to cracking and slow healing. Immune system function can also be affected, including an altered inflammatory process [7, 8].

The aim of this retrospective study, which was conducted at the Concord Hospital Burns Unit, was to compare differences in outcomes for patients with foot burns with and without diabetes. Examining the differences in outcome measures, including age, relevant demographic data, hospital length of stay and number of surgeries required, can assist health professionals to identify patients potentially at risk of poorer outcomes. These patients may require altered methods of care to achieve optimum results in healing and to prevent complications leading to longer hospital stays. Identifying population groups at high risk of avoidable burns may also emphasize the need for more targeted burns prevention education.

## Methods

A retrospective notes audit was conducted for all patients with foot burns admitted to the Concord Hospital Burns Unit from 1^st^ January 2012 to 31^st^ December 2013. The Agency of Clinical Innovation Database and the Concord Hospital Burns Unit admissions records were used to identify 99 subjects with foot burns, including 15 with diabetes and 84 without diabetes. Data were collected for all 15 subjects with diabetes and foot burns, and 18 subjects with foot burns but without diabetes were randomly selected as a control group. Subjects were matched for percentage total body surface area of burns; the median was 2.00 % (interquartile range, IQR, 1.00–3.30 %) in the diabetic group and 1.75 % (IQR, 1.0–4.25 %) in the control. Demographic data and various outcome measures were recorded including age, gender, working status, inpatient and outpatient length of stay, size of burn, grafting, re-grafting, amputations and prescription of compression garments.

Ethics approval was granted for a notes audit by the Concord Hospital Human Research and Ethics Committee.

### Statistical analysis

For continuous variables, a normal distribution test was first performed. If the indices followed normal distributions, they are expressed as the means and the 95 % confidence interval (95 % CI), and a *t*-test was performed. Non-continuous variables are shown as median and IQR, and the Mann–Whitney *U* test was performed. For categorical variables, a Chi-square test and Fisher’s Exact Test were performed because the total sample size was only 33, and thus the expected count may be less than 5. Statistical analysis was performed with SPSS (version 22.0), and a two-tailed probability value of less than 0.10 was considered statistically significant.

## Results

**Fig. 1:**
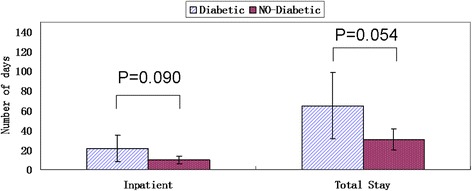
Histogram and comparisons of inpatient and total stay in diabetes and control groups. The error bars indicate a 95 % confidence interval (CI) for each mean (histograms)

**Fig. 2:**
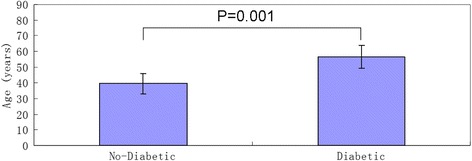
Histogram and comparisons of age in diabetes and control groups. The error bars indicate a 95 % confidence interval (CI) for each mean (histograms)

Results show that patients with diabetes and foot burns have a significant difference in outcomes. The mean inpatient and total length of stay for the diabetic group were 21.27 (95 % CI, 7.9472–34.5862) days and 64.80 (95 % CI, 31.1270–98.4730) days, which were significantly longer (*P* = 0.090 and *P* = 0.054) than 9.61 (95 % CI, 5.7966–13.4257) days and 30.56 (95 % CI, 19.7784–41.3328) days in the control group (Fig. 1), based on the 0.10 significance level. The diabetic group was significantly older (*P* = 0.001) at 56.6000 (95 % CI, 49.3559–63.8441) years versus 39.4444 (95 % CI, 32.8732–46.0157) years in the control group (Fig. 2).

**Fig. 3:**
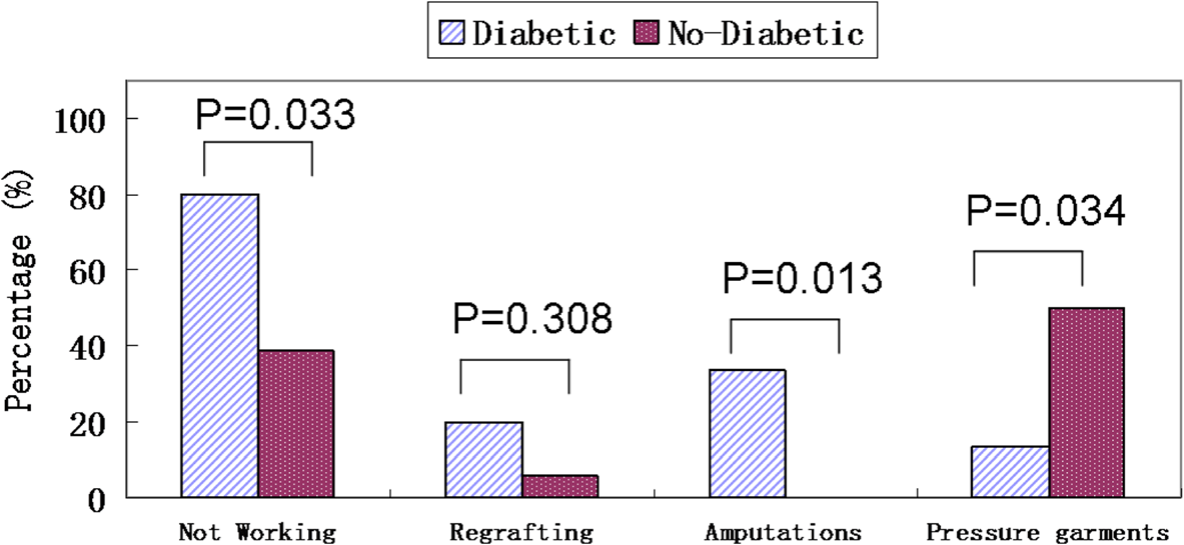
Histogram and comparisons of outcome measures in diabetes and control groups

For the outcome measures, significantly (*P* = 0.033) more patients with diabetes were not working (*n* = 12/15 or 80.00 % versus *n* = 7/18 or 38.89 %) compared to the control group. The diabetic group had higher rates of regrafting (*n* = 3/15 or 20 % versus *n* = 1/18 or 6 %) than the control group and significantly (*P* = 0.013) more amputations (*n* = 5/15 or 33.33 % versus *n* = 0 or 0.00 %) compared to the control group. Fewer patients with diabetes were prescribed pressure garments (*n* = 2/15 or 13.33 % versus *n* = 9/18 or 50.00 %), which was significant (*P* = 0.034). See Fig. 3.

## Discussion

There are multiple management options for patients with diabetic foot burns. Conservative management requires a long healing time and places considerable demands on health services. Surgical approaches also have risks because results indicate more amputations and regrafts were required in the diabetic group. Healing time may be reflected by total length of stay, which is more than double for patients with diabetes, increasing demand and cost of inpatient and outpatient services [1, 8].

The finding of increased age of the diabetic group correlates with results from other studies [1, 9]. Increased age has been shown to contribute to poorer outcomes when adjusted for injury severity, comorbidities and complications [10]. Kimball et al report that age itself is not a cause of increased complications with burns; rather, it is the age-associated increased risk of comorbidities which contributes to increased morbidity and mortality in patients with diabetes and foot burns [1]. Additionally, it has been shown that patients with diabetes still have worse outcomes, including increased length of stay and more skin grafts and infections compared to patients without diabetes when matched for age and size of burn [8].

In our study, the group with diabetes were less likely to be working, required more regrafts and amputations and were less likely to be prescribed pressure garments. The fact that they were less likely to be working may reflect a difference in socioeconomic status because diabetes has been shown to be more prevalent in people who are retired or unemployed compared with people who are in paid work [11]. The association between diabetes and socioeconomic status has also been shown to remain statistically significant when accounting for modifiable lifestyle factors such as smoking, obesity and physical activity [11]. The higher rate of regrafting and amputations in the group with diabetes is consistent with findings in other studies [2, 8, 12]. The pathophysiology of impaired wound healing in diabetes is well documented [7, 8, 12]. Vascular insufficiency, peripheral neuropathy and altered immune function all contribute to poorer outcomes in patients with diabetes. Finally, pressure garments for patients with diabetes can be prescribed for scar management; however, results indicate this was not suitable for many patients with diabetes. Risks associated with pressure garments used for management of scarring include wound breakdown from friction or pressure points [13]. A patient with diabetes and altered vascularity or neuropathy would require close monitoring and evaluation of the skin to maintain adequate blood flow and prevent wound breakdown.

A limitation of this study is that the control group was randomly selected and does not have an equal number of subjects compared to the diabetes group. The results may be more reliable if all control subjects were included in the comparison of outcome measures with the diabetic group. In addition, current data were drawn from 2012 to 2013. Burn management in general undergoes constant modification. Over time, the management of diabetic foot burns changed from aggressive surgery to a more conservative approach. Perhaps a larger database with a larger sample size can provide a more accurate reflection of post-burn outcomes in people with diabetes.

## Conclusion

Patients with diabetes and foot burns have an altered pathway of care and a higher rate of complications with healing, reflected by a total length of stay that is more than double the average of that for patients without diabetes. The diabetic group was older and less likely to be working. Patients with diabetes required more regrafting and amputations and had a lower rate of pressure garment prescription. This study highlights the importance of recognizing the potential for poorer outcomes for patients with diabetes and foot burns and indicates the need for more burns prevention education and promotion in this at risk patient group.
